# A U-Box E3 Ubiquitin Ligase, PUB20, Interacts with the Arabidopsis G-Protein β Subunit, AGB1

**DOI:** 10.1371/journal.pone.0049207

**Published:** 2012-11-15

**Authors:** Shio Kobayashi, Daisuke Tsugama, Shenkui Liu, Tetsuo Takano

**Affiliations:** 1 Asian Natural Environmental Science Center (ANESC), The University of Tokyo, Tokyo, Japan; 2 Alkali Soil Natural Environmental Science Center (ASNESC), Northeast Forestry University, Harbin, People’s Republic of China; University of Minnesota, United States of America

## Abstract

An Arabidopsis U-box E3 ubiquitin ligase Plant U-box 20 (PUB20; alternatively called AtCMPG1) was identified as a possible interactor of the Arabidopsis G-protein β subunit, AGB1, by yeast two-hybrid screening. A bimolecular fluorescence complementation (BiFC) assay showed that PUB20 interacted with AGB1 in the nuclei and the cytosol. The expression levels of PUB20 and its closest homolog, PUB21 were stable under many conditions. *GUS* driven by the PUB20 promoter was active in anthers, pollen, premature seeds and receptacles and *GUS* driven by the PUB21 promoter was active in anthers and funiculi. PUB20 was found to have autoubiquitination activity *in vitro*.

## Introduction

Heterotrimeric G-proteins (G-proteins) are evolutionarily conserved plasma membrane-bound proteins that are involved in intracellular signaling. G-proteins consist of α, β and γ subunits. G-protein signaling has been extensively studied in yeast and mammals, but is not well understood in plants. Mammals have 23 Gα, 5 Gβ, and 12 Gγ, but plants have a much simpler system and only 1 Gα, 1 Gβ and 3 Gγs have been identified in *Arabidopsis thaliana* [1], [2]. However, despite the simplicity of the system, G-proteins are involved in many biological processes.

Gβ of Arabidopsis is named AGB1. *agb1*, the loss-of-function mutant of AGB1, shows morphological aberrations [3], [4], an etiolated and light-grown phenotype under dark conditions [5], increased stomatal density [6], altered phytohormone responsiveness [7], [8] and reduced responsiveness to pathogens [9]. Although the roles of AGB1 in plants are becoming clearer, the downstream effectors of AGB1 and other components of the AGB1 signaling pathways remain largely unknown. We conducted yeast two-hybrid screening to identify proteins which interact with AGB1, and identified an E3 ubiquitin ligase Plant U-box 20 (PUB20; otherwise known as AtCMPG1 for CYS, MET, PRO, and GLY protein 1) as a possible interaction partner.

PUB20 belongs to the U-box E3 ubiquitin ligase family whose members have a ∼70 amino acid U-box motif. PUB20 is predicted to have a U-box at its N-terminus and another motif called Armadillo (ARM) repeats at the C-terminus [10]. PUB20 is upregulated by PAMP treatment and by infection of *Pseudomonas syringae*, and wounding [11]. A microarray analysis revealed that PUB21, the closest homolog of PUB20, is also induced by PAMP and pathogen treatment as well as PUB20 [12]. The amino acid sequences of PUB20 and PUB21 share 56% identity and 72% similarity. In this report, we show the interaction of PUB20 with AGB1, the expression analysis of PUB20 and 21 and the autoubiquitination activity of PUB20 *in vitro*.

**Figure 1:**
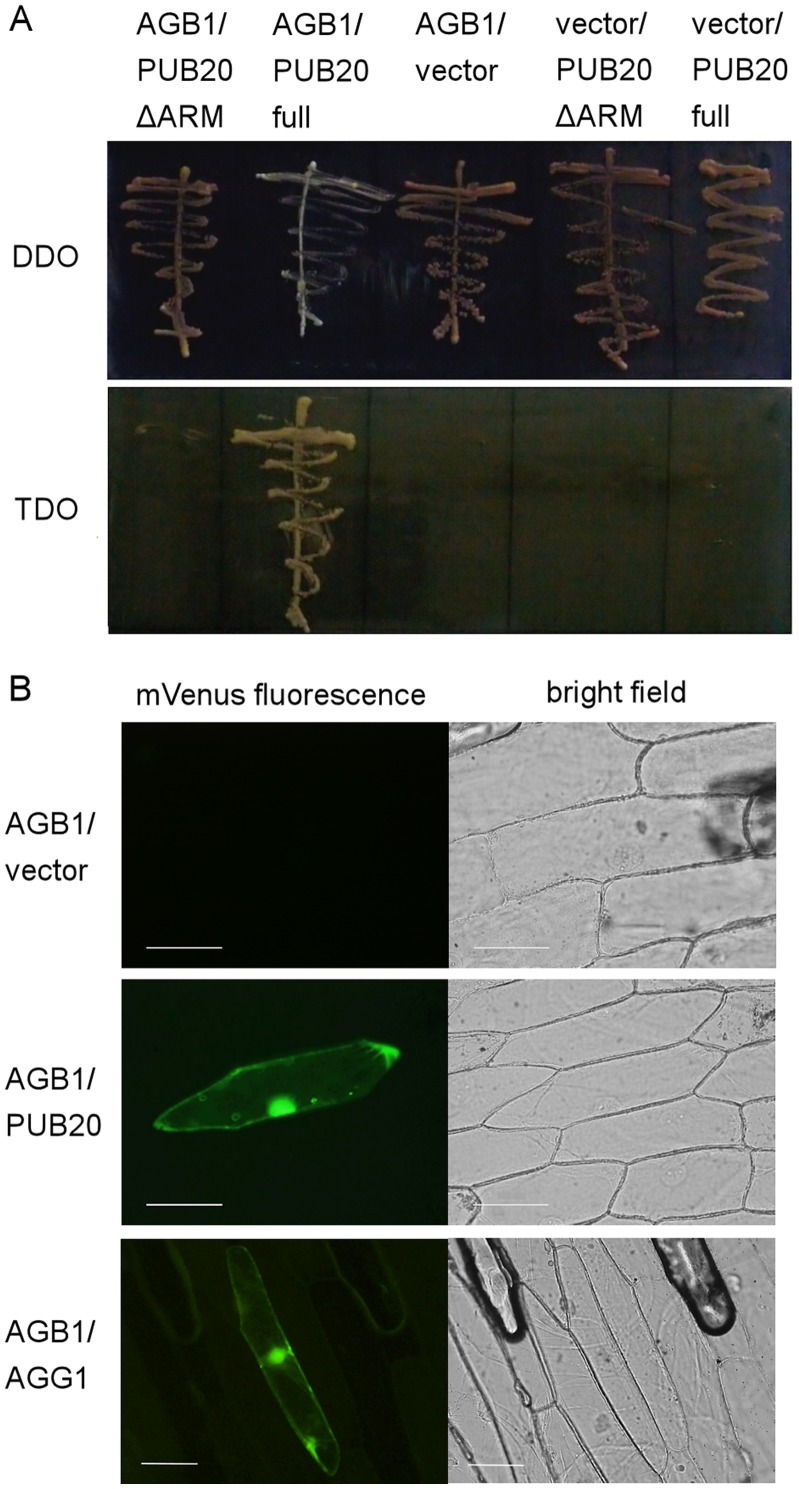
Interaction between PUB20 and AGB1. (A) Yeast two-hybrid assay. The combinations of the plasmids used for transformation of the yeast strain AH109 are indicated at the top of the panel (bait/prey). vector: a pGBKT7 plasmid or a pGADT7-Rec plasmid containing no insert, AGB1: pGBKT7-AGB1, PUB20: pGADT7-Rec-PUB20 (full-length ORF), PUB20ΔARM: pGADT7-Rec-PUB20 (truncated ORF without ARM repeats). Yeast cells were cultured on DDO (SD/−Trp/−Leu; control) and TDO (SD/−Trp/−Leu/−His+10 mM 3-AT) plates to check activation of the reporter gene, *HIS3*. (B) Bimolecular fluorescence complementation (BiFC) assay. The florescences in the cytoplasm (left) indicate complementation of the fluorescence of mVenus by interaction between PUB20 or AGG1 (positive control) [21] and AGB1. The combinations of the plasmids introduced are shown in the left (VN/VC). AGB1: pBS-35S:AGB1-VN159, PUB20: pBS-35S:PUB20-VC80, vector: pBS-35S:VC80, AGG1: pBS-35S:AGG1-VC80. mCherry was introduced together with negative controls to confirm that the cell was successfully transformed (data not shown). Scale bars = 100 µm.

## Materials and Methods

### Yeast Two-hybrid Screening

Yeast two-hybrid experiments were performed using the Matchmaker Two-Hybrid System (Clontech). A cDNA library for the yeast two-hybrid screen was prepared using mRNA samples from *Arabidopsis* mature leaves. Full-length cDNA clones of AGB1 (RAFL05-19-B08) was obtained from the RIKEN BRC Experimental Plant Division. The open reading frame (ORF) of AGB1 was amplified by PCR and cloned into pGBKT7 (pGBK-AGB1). pGBK-AGB1 was used as a bait for the yeast two-hybrid screen. After the screen by yeast mating, the PUB20 ORF was amplified by yeast colony PCR from yeast cells expressing PUB20, and cloned in-frame behind the GAL4 activation domain (GAL4AD) of pGADT7-Rec (pGAD-PUB20). The truncated form of PUB20 lacking ARM repeats (PUB20ΔARM) was amplified using the primer pair shown in Table S1 and pGAD-PUB20 as a template, and was inserted into the pGADT7-Rec plasmid. pGBK-AGB1 and each of the above pGAD constructs were co-introduced into the *Saccharomyces cerevisiae* strain AH109. After transformation, at least 4 colonies grown on the DDO (SD/−Leu/−Trp) agar plates were streaked on the TDO (SD/−Leu/−Trp/−His) and QDO (SD/−Leu/−Trp/−His/−Ade) agar plates to check the interactions between the proteins of interest.

**Figure 2:**
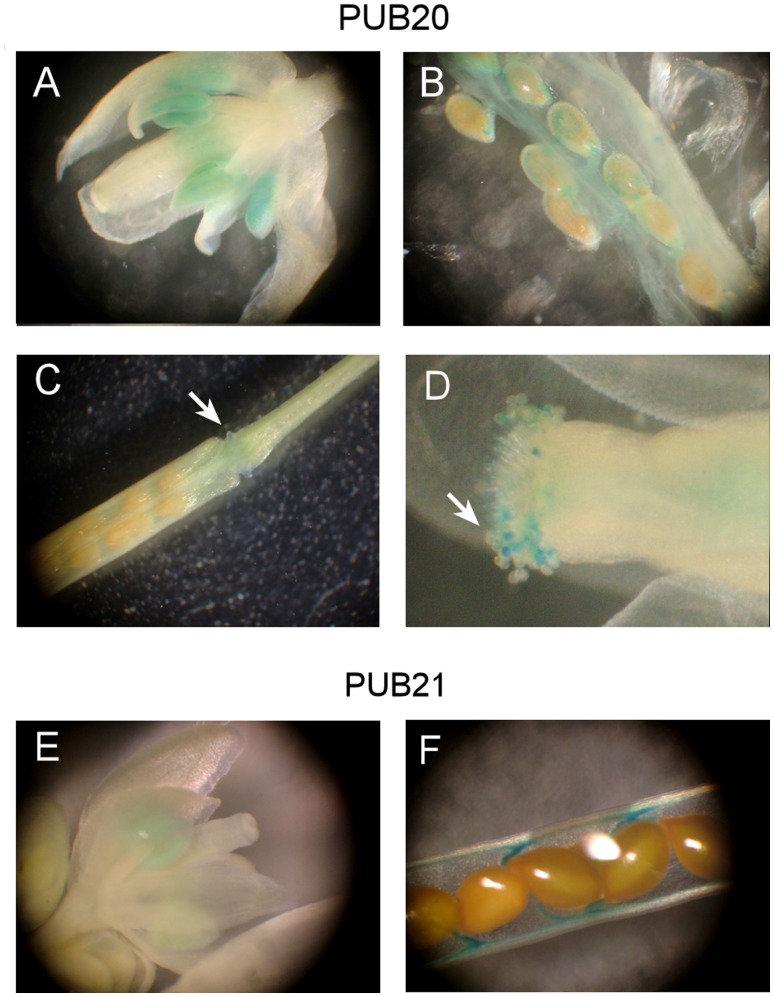
Promoter:*GUS* analysis. T_2_ generations of Arabidopsis plants expressing PUB20 promoter:*GUS* (A-D) or PUB21 promoter:*GUS* (E, F) were analyzed. GUS activity was observed at anthers (A), premature seeds and in funiculi (B), receptacles (C) and in pollens (D) in PUB20 promoter:*GUS* transgenic lines. GUS activity was observed in anthers (E) and in funiculi (F) in PUB21 promoter:*GUS* transgenic lines. Arrowheads mark regions of GUS staining.

### Bimolecular Fluorescence Complementation (BiFC) Assay

The vector for BiFC assay was constructed by replacing GFP in the vector pBS-35SMCS-GFP [13] with the N-terminus (154 aa) or the C-terminus (80 aa) of mVenus. The full-length ORFs of *AGB1*, *AGG1* and *PUB20* were amplified using the primer pairs shown in Table S1 and were inserted into the vector containing the N-terminus (*VN154*; for *AGB1*) or the C-terminus (*VC80*; for *AGG1* and *PUB20*) of *mVenus*, respectively. Transient expression of the resultant constructs and the observation of fluorescence were performed as previously described [14].

**Figure 3:**
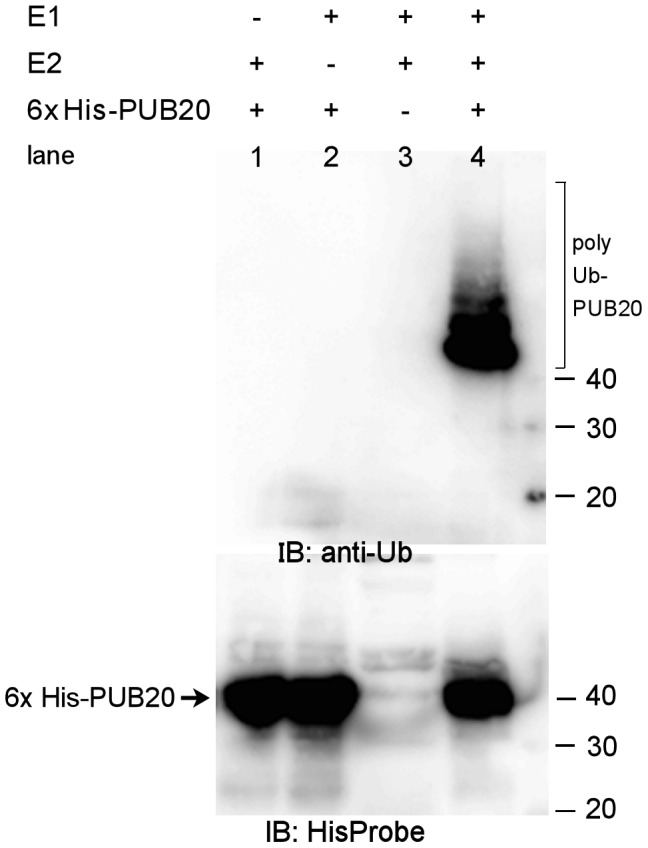
In vitro ubiquitination assay. Recombinant 6× His-PUB20 protein was expressed using *E. coli* and incubated at 28°C for 1 h in the presence or absence of E1 (UBA1) and E2 (UbcH5b). The samples were separated by 7.5% SDS-PAGE and subjected to immunoblot analysis by anti-Ub antibody (top) and HisProbe (bottom). The numbers in the right indicate molecular sizes (kDa). The part of the blot showing free Ub (8.6 kDa) was cut off.

### Expression Analyses

Surface-sterilized Arabidopsis seeds (ecotype: Col-0) were sown on 0.8% w/v agar plates containing 0.5× MS salts, 0.735% w/v sucrose and Gamborg’s vitamin solution, pH 5.7, and were germinated at 4°C in the dark for 48 h. Plants were grown at 22°C under 16 h-light/8 h-dark condition (light intensity 120 µmol⋅m^−2^⋅s^−1^).

For semi-quantitative PCR, two-week-old plants were transplanted to 1/4× MS liquid medium (pH 5.7) and allowed to grow for another one or two weeks, and subjected to stress treatments. For cold treatment, four-week-old plants on liquid medium were put into a refrigerator and kept at 4°C for 5 or 10 h. For ABA or NaCl treatment, four-week-old plants were transferred to 1/4× MS liquid media containing 100 µM ABA, 300 mM NaCl, ethanol (mock treatment for ABA) or DW (mock treatment for NaCl), respectively, and sampled after 1.5 and 3 h. For drought treatment, four-week-old plants were placed on filter paper and kept at 22°C for 0.5 or 1 h. For flg22 treatment, three-week-old plants were incubated in 20 mM Tris-HCl (pH 6.8) containing 1 µM flg22 for 0.5 or 1 h. For *Agrobacterium* treatment, three-week-old plants were incubated in the DW containing *Agrobacterium tumefaciens* (OD_600_ = 1.0) for one minute and then incubated on 1/2× MS plates for 3, 6 or 12 h. For growth stage- or organ-specific expression analysis, 10-d-old plants grown on a 1/2× MS agar plate (for seedlings) or plants grown on a 1/2× MS agar plate for two weeks and subsequently on 1/4× MS liquid medium for another four weeks (for mature leaves, roots, flowers, stems) were sampled. mRNA was extracted from shoots (for stress treatments) or each organs (for organ-specific sampling) by guanidium isothiocyanate (GTC) method and reverse transcribed with PrimeScript Reverse Transcriptase (Takara Bio) using an oligo (dT) primer. RT-PCR was performed using primer pairs shown in Table S2 and GoTaq Green Master Mix (Promega).

For promoter:*GUS* analysis, the promoter regions of PUB20 (−1326∼0 bp) and PUB21 (−1740∼0 bp) were amplified using the primer pairs shown in Table S1, using Arabidopsis genomic DNA as a template. The amplification products were inserted upstream of the *GUS* gene in the pBI121 vector. The resultant plasmids were introduced into Arabidopsis (ecotype: Col-0) by the floral dip method, using *Agrobacterium* strain EHA105 as previously described [15]. Plants from the T_2_ generation were analyzed for β-glucuronidase (GUS) expression.

### In vitro Ubiquitination Assay

For expression of recombinant proteins in *E. coli*, the full-length coding region of PUB20 was transferred from the pGADT7-Rec-PUB20 plasmid to the pET-32a (+) vector and fused in-frame with a 6× His-tag at the N-terminus. The resultant plasmid was transformed into the *E. coli* strain BL21 (DE3). The recombinant proteins were expressed and purified as previously described [13]. Autoubiquitination was assayed as previously described [16]. Five µl of the concentrate of 6× His-PUB20 was incubated at 28°C in the presence or absence of ubiquitin activating enzyme E1 (UBA1), ubiquitin conjugating enzyme E2 (UbcH5b), ubiquitin (Ub) and ATP for 1 h and then subjected to immunoblot analysis using anti-Ub antibody (P4D1) or HisProbe-HRP.

### Characterization of Pub20 Mutant

A T-DNA insertion mutant of PUB20 was obtained from Arabidopsis Bioresource Center (stock no. SAIL_240_C09). According to the database (The Arabidopsis Information Resource; http://www.arabidopsis.org/), the *pub20* mutant has a T-DNA insertion at around 770 bp downstream of the initiation codon (Figure S4A). Genomic DNA was extracted from 6-d-old seedlings of wild-type (WT; Col-3) and the *pub20* mutant plants by a chemical method previously described [17] and T-DNA insertion was confirmed by genomic PCR. The *PUB20* ORF-specific primers used for the PCR are described in Figure S4 and the primer sequences are shown in Table S3. The sequence of the T-DNA-specific primer LB3 (5′-TAG CAT CTG AAT TTC ATA ACC AAT CTC GAT ACA C-3′) was obtained from the website of The Nottingham Arabidopsis Stock Centre (NASC; http://arabidopsis.info/). The PCR was performed using KOD FX Neo (TOYOBO). The expression of *PUB20* mRNA in the WT and the *pub20* mutant was tested by RT-PCR. Total RNA was prepared using GTC method and cDNA was synthesized with PrimeScript Reverse Transcriptase (Takara Bio) using an oligo (dT) primer. The primers used for the RT-PCR are shown in Figure S4 and the primer sequences are described in Table S2 and S3. Surface-sterilized seeds (Col-3 and *pub20*) were germinated on 0.5× MS agar plates (described in “Expression analysis” section) with or without 1 µM ABA, 10 ppb brassinolide, 100 mM NaCl, 1µM flg22 or 5µM brassinazole (brassinosteroid biosynthesis inhibitor), and the germination rates and later growth on the plates were compared in the same condition as described in “Expression analysis” section.

## Results and Discussion

### PUB20 Interacts with AGB1 in the Nuclei and the Cytosol

To identify potential interactors of AGB1, we performed a yeast two-hybrid screen of the *Arabidopsis* leaf library using full-length AGB1 as bait. Even on high-stringency selection media (SD/QDO), more than 3600 positive clones were obtained. Using yeast colony PCR with an *AGG1*- or *AGG2*-specific primer, we found that 60–70% of these clones expressed AGG1. Plasmid inserts from non-*AGG1* clones were then amplified by colony PCR using a vector-specific primer pair, and sequenced. Around 400 clones were sequenced, and 14 of them expressed PUB20. Figure 1A shows the result of the yeast two-hybrid assay. To identify the region of PUB20 interacting with AGB1, we amplified the truncated form of PUB20 lacking putative ARM repeats (PUB20ΔARM) and used it as a prey along with full-length PUB20. PUB20ΔARM is described in Figure S1. The result showed that PUB20 interacts with AGB1 in an ARM repeats-dependent manner. The interaction of full-length PUB20 with AGB1 was confirmed by a bimolecular fluorescence complementation (BiFC) assay (Figure 1B). The result showed the interaction of PUB20 and AGB1 in the nuclei and the cytoplasm. Drechsel *et al*. reported that PUB20-GFP fusion protein was localized in the cytoplasm [18]. Also, our observation showed that AGB1 is present in the cytoplasm (Figure S2), as well as nuclei and the plasma membrane as reported by Anderson and Botella [19]. AGB1 is already known to interact with an E3 ubiquitin ligase, DDB1 [20], but the location and the purpose of the interaction has not been shown. The function of AGB1 in the cytosol is unknown. It is possible that the internalization into the cytosol and subsequent degradation by E3 ubiquitin ligases is responsible for regulating AGB1, although it is unclear whether these E3 ligases are the effectors or regulators of AGB1. PUB21 was localized in the cytoplasm (Figure S2), and it did not interact with AGB1 in the yeast two-hybrid assay (data not shown). The interaction between AGG1 and AGB1 is shown as a positive control [21].

### Expression of PUB20 and PUB21

For both PUB20 and PUB21, the expression levels were similar in seedlings, mature leaves, roots, flowers and stems, and were not altered in shoots by various biotic (*Agrobacterium* and flg22) or abiotic (chilling, NaCl and drought) stress treatments (Figure S3). PUB20 responded to wounding (Figure S4) as previously reported by PUB20 promoter:*GUS* staining [11] and microarray analysis [12]. However, in contrast to previous findings [11], [12], PUB20 expression was not increased by an elicitor (flg22) or a pathogen (*Agrobacterium tumefaciens*) (Figure S3B, C). This discrepancy may be due to the difference in the tissue used for mRNA extraction (parsley protoplast in Heise’s report vs. Arabidopsis whole plant in our experiment). These results suggest that PUB20 has no critical role in the response to these stress conditions.

The GUS staining patterns of the PUB20 promoter:*GUS* lines and PUB21 promoter:*GUS* lines are shown in Figure 2. PUB20 was found to be transcribed in receptacles (Figure 2C), as reported previously [11], and in anthers, mature pollen, premature seeds and funiculi (Figure 2A, B, D). PUB21 was expressed in anthers and funiculi (Figure 2E, F).

These results show that PUB20 has a low basal expression level throughout the plant, and that this is specifically induced in some floral organs or by wounding. *GUS* driven by AGB1 promoter was active throughout Arabidopsis plants including anthers and receptacles [19], suggesting that the interaction between PUB20 and AGB1 has a role in these floral organs, although further study is needed to interpret these results.

### In vitro Ubiquitination Assay

Although the deduced amino acid sequence of PUB20 suggested that it was an E3 ubiquitin ligase, it was unclear whether it had E3 ubiquitin ligase activity. To show activity, E3 ubiquitin ligases require both a ubiquitin-activating enzyme E1 (which activates ubiquitin so it can be attached to substrate proteins) and a ubiquitin-conjugating enzyme E2 (which attaches ubiquitins to substrate proteins in cooperation with E3) in addition to ATP and ubiquitin (Ub). Immunoblotting by anti-Ub antibody showed that PUB20 was ubiquitinated when it was reacted in the presence of both E1 and E2 (Figure 3, upper panel, lane 4) but not in the absence of either or both of E1 and E2 (lanes 1–3). A ubiquitin ladder can be seen in the region labeled poly Ub-PUB20. Immunoblotting by the anti-His probe (bottom panel) confirms that the main band is PUB20. These results clearly show that PUB20 has E3 ubiquitin ligase activity. PUB20 did not ubiquitinate AGB1 or decrease its level (data not shown), which suggests PUB20 is modulated by AGB1, rather than vice versa. However, further study is needed to confirm this hypothesis.

In an attempt to characterize further the function of PUB20, we obtained a mutant of PUB20 that has a T-DNA insertion at around 770 bp downstream of the initiation codon (Figure S4A). Genomic PCR analysis was performed to confirm the T-DNA insertion. The primer pair of a T-DNA-specific (LB3 in Figure S4A) and a *PUB20* ORF-specific (RV2 in Figure S4A) primers yielded a band only in the *pub20* mutant and not in WT (Col-3), whereas the pair of *PUB20* ORF-specific primers (FW2 and RV2 in Figure S4) with extension time of 30 seconds yielded a band only in WT and not in the *pub20* mutant (Figure S4B), suggesting that the mutant has a T-DNA insertion in the predicted region (Figure S4B). In RT-PCR analyses, the primer pair designed to amplify the region upstream of the T-DNA insertion (Figure S4A, FW1 & RV1) yielded products for both the *pub20* mutant and WT (Col-3), while the primer pair designed to amplify the region downstream of the T-DNA insertion (Figure S4A, FW2 & RV2) yielded a product only for the WT. These results suggest that the mutant truncated PUB20 mRNA, which is missing the 3′ region following the T-DNA insertion, is expressed (Figure S4C). Because the ARM repeat domain of PUB20 is required for the interaction with AGB1 (Figure 1A), we did not expect the truncated PUB20 protein to interact with AGB1. Treating the *agb1* mutant with ABA or brassinolide alters the germination rates compared with WT [3], [8] (Table S4). However, these treatments had similar effects on *pub20* mutants and the WT (Table S4). In addition, 100 mM NaCl, 1 µM flg22, 5 µM brassinazole (brassinosteroid biosynthesis inhibitor) had similar effects on germination rates and the subsequent growths of *pub20* mutant and WT (Table S4). Further studies are needed to determine the roles of PUB20 in AGB1-mediated intracellular signaling.

## Supporting Information

Figure S1 **The primary sequences of full-length PUB20 protein and PUB20ΔARM protein.** Solid underlines indicate the U-box and ARM repeats identified by Trujillo, Ichimura, Casais and Shirasu (Current Biology 18∶1396-1401, 2008). Dotted underline indicates the region of PUB20 used as PUB20ΔARM (Fig. 1A). Identical and similar residues are shown in black and gray, respectively.
(PDF)

Figure S2 **Subcellular localizations of PUB21-GFP and AGB1-GFP fusion proteins in onion epidermal cells.** Scale bars = 100 µm.
(PDF)

Figure S3 **Expression analysis of PUB20 and PUB21 by semi-quantitative RT-PCR.** **(A)** Four-week-old plants of Arabidopsis (ecotype: Col-0) were subjected to low temperature (4°C), 100 µM ABA, 300 mM NaCl or drought stress treatment before sampling. Drought stress treatment was performed by placing the plants on a filter paper and keeping them in the growth chamber set at 22°C, and other stress treatments were performed on liquid media. **(B)** Three-week-old Arabidopsis plants were incubated in 20 mM Tris-HCl (pH 6.8) containing 1 µM flg22 for indicated times. **(C)** Three-week-old Arabidopsis plants were incubated in the DW containing *Agrobacterium tumefaciens* (OD_600_ = 1.0) for one minute and then incubated on 1/2× MS plates for indicated times. **(D)** 10-d-old (for seedlings) or five to six-week-old (for mature leaves, roots, flowers, stems) Arabidopsis plants were sampled. Primer pairs used are listed in Table S2.
(PDF)

Figure S4 **pub20 mutant. (A)** A schematic representation of *pub20* allele, with the T-DNA insertion shown as an inverted triangle. Primer pairs used for genomic PCR analysis (B) and RT-PCR analysis (C) are shown with arrows. The U-box domain (black box) and the ARM repeat domain (gray box) were predicted by comparing the amino acid sequence of PUB20 with PUBs described by Trujillo, Ichimura, Casais and Shirasu (Current Biology 18∶1396-1401, 2008). **(B)** Confirmation of T-DNA insertion in the *pub20* mutant by genomic PCR. The sequences of primers specific to *PUB20* ORF (FW2 and RV2) is shown in Table S3. The sequence of the T-DNA-specific primer LB3 was obtained from the website of The Nottingham Arabidopsis Stock Centre (NASC; http://arabidopsis.info/). **(C)** Expression analysis of *UBQ5* and *PUB20* by RT-PCR. Primers are listed in Table S2 and S3.
(PDF)

Table S1 **Primers used for making constructs in this work.**
(PDF)

Table S2 **Primers used in real-time RT-PCR analysis.**
(PDF)

Table S3 **Primers used in genotyping and RT-PCR analysis of the pub20 mutant (Fig. S4B, C).**
(PDF)

Table S4 **Effects of plant hormones on germination rate.**
(PDF)
