# Supplementary material for: A U-Box E3 Ubiquitin Ligase, PUB20, Interacts with the Arabidopsis G-Protein β Subunit, AGB1
Source: PLoS One. 2012 Nov 15;7(11):e49207. doi: 10.1371/journal.pone.0049207 (PMC3499536; doi:10.1371/journal.pone.0049207)
Supplement: Figure S1 — The primary sequences of full-length PUB20 protein and PUB20ΔARM protein. Solid underlines indicate the U-box and ARM repeats identified by Trujillo, Ichimura, Casais and Shirasu (Current Biology 18∶1396-1401, 2008). Dotted underline indicates the region of PUB20 used as PUB20ΔARM (Fig. 1A). Identical and similar residues are shown in black and gray, respectively. (PDF) [file pone.0049207.s001.pdf]

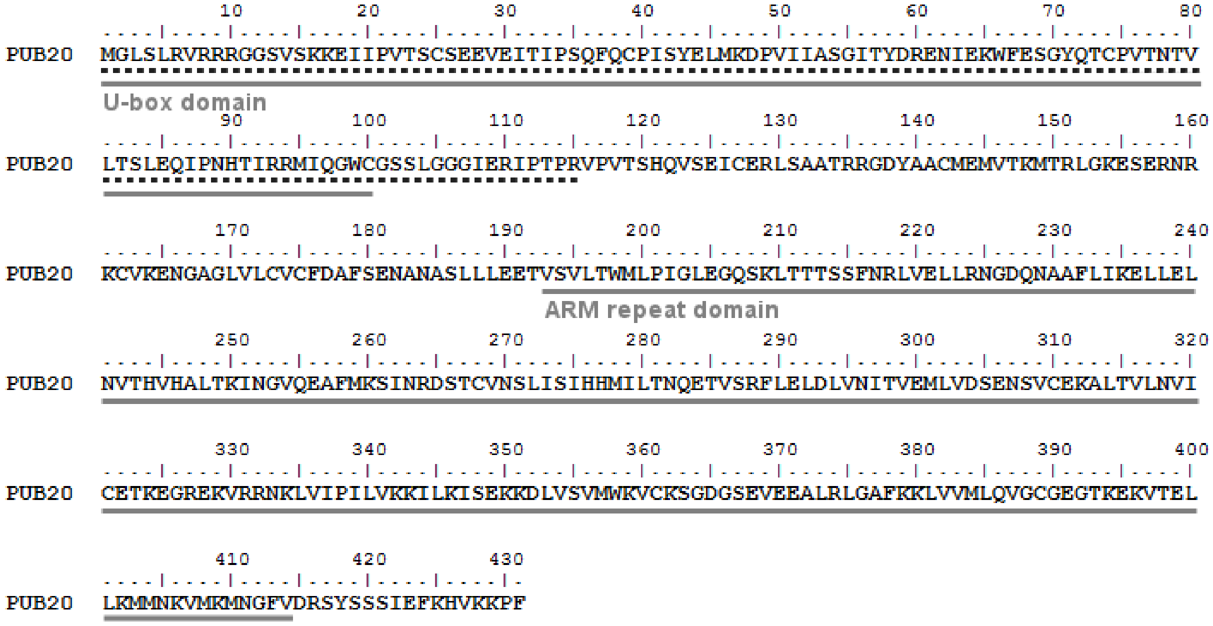

Figure S1. The primary sequences of full-length PUB20 protein and PUB20ΔARM protein. Solid underlines indicate the U-box and ARM repeats identified by Trujillo, Ichimura, Casais and Shirasu (Current Biology 18: 1396-1401, 2008). Dotted underline indicates the region of PUB20 used as PUB20ΔARM (Fig. 1A). Identical and similar residues are shown in black and gray, respectively.
