# Supplementary material for: A U-Box E3 Ubiquitin Ligase, PUB20, Interacts with the Arabidopsis G-Protein β Subunit, AGB1
Source: PLoS One. 2012 Nov 15;7(11):e49207. doi: 10.1371/journal.pone.0049207 (PMC3499536; doi:10.1371/journal.pone.0049207)
Supplement: Figure S2 — Subcellular localizations of PUB21-GFP and AGB1-GFP fusion proteins in onion epidermal cells. Scale bars = 100 µm. (PDF) [file pone.0049207.s002.pdf]

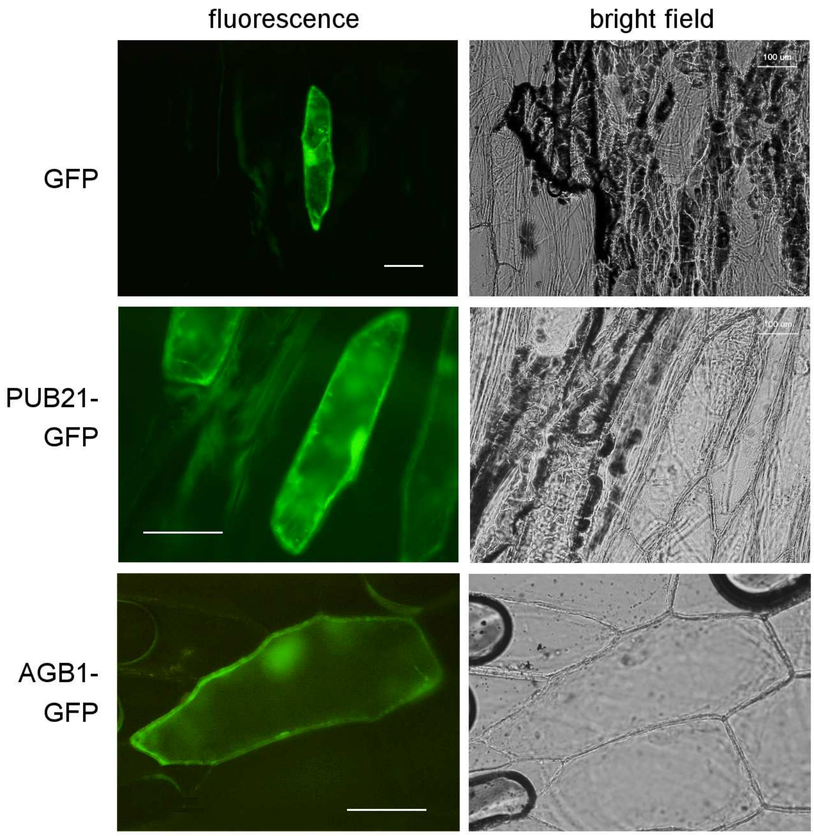

Figure S2. Subcellular localizations of PUB21-GFP and AGB1-GFP fusion proteins in onion epidermal cells. Scale bars = 100 µm.
