# Supplementary material for: A U-Box E3 Ubiquitin Ligase, PUB20, Interacts with the Arabidopsis G-Protein β Subunit, AGB1
Source: PLoS One. 2012 Nov 15;7(11):e49207. doi: 10.1371/journal.pone.0049207 (PMC3499536; doi:10.1371/journal.pone.0049207)
Supplement: Figure S3 — Expression analysis of PUB20 and PUB21 by semi-quantitative RT-PCR. (A) Four-week-old plants of Arabidopsis (ecotype: Col-0) were subjected to low temperature (4°C), 100 µM ABA, 300 mM NaCl or drought stress treatment before sampling. Drought stress treatment was performed by placing the plants on a filter paper and keeping them in the growth chamber set at 22°C, and other stress treatments were performed on liquid media. (B) Three-week-old Arabidopsis plants were incubated in 20 mM Tris-HCl (pH 6.8) containing 1 µM flg22 for indicated times. (C) Three-week-old Arabidopsis plants were incubated in the DW containing Agrobacterium tumefaciens (OD600 = 1.0) for one minute and then incubated on 1/2× MS plates for indicated times. (D) 10-d-old (for seedlings) or five to six-week-old (for mature leaves, roots, flowers, stems) Arabidopsis plants were sampled. Primer pairs used are listed in Table S2. (PDF) [file pone.0049207.s003.pdf]

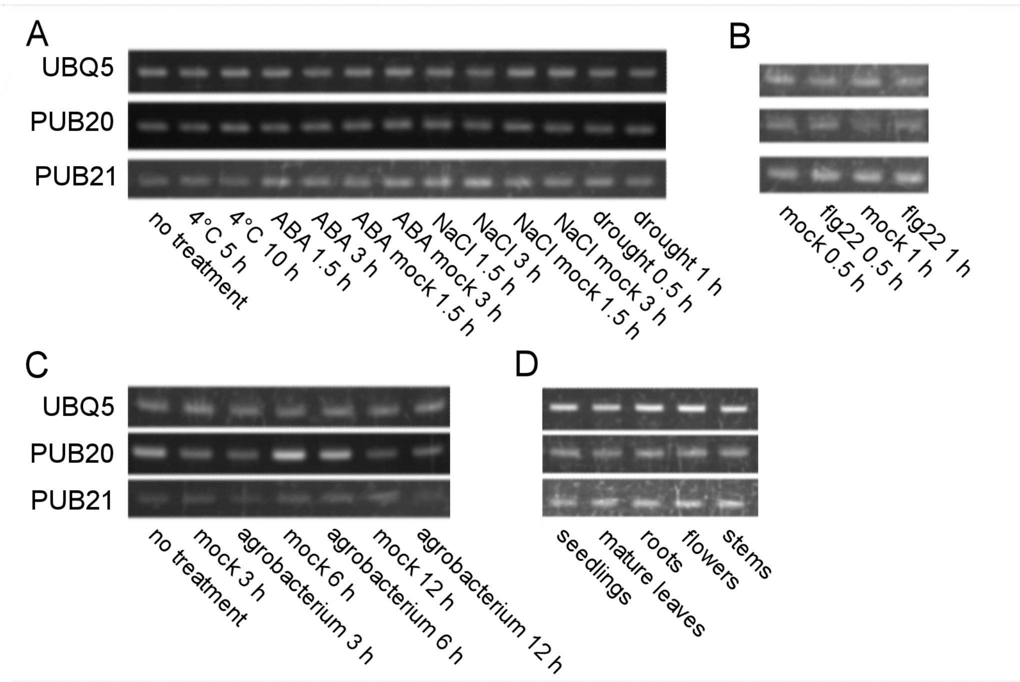

Figure S3. Expression analysis of PUB20 and PUB21 by semi-quantitative RT-PCR. (A) Four-week-old plants of Arabidopsis (ecotype: Col-0) were subjected to low temperature (4 °C), 100 μM ABA, 300 mM NaCl or drought stress treatment before sampling. Drought stress treatment was performed by placing the plants on a filter paper and keeping them in the growth chamber set at 22 °C, and other stress treatments were performed on liquid media. **(B)** Three-week-old Arabidopsis plants were incubated in 20 mM Tris-HCl (pH 6.8) containing 1 μM flg22 for indicated times. **(C)** Three-week-old Arabidopsis plants were incubated in the DW containing *Agrobacterium tumefaciens* (OD_{600} = 1.0) for one minute and then incubated on 1/2x MS plates for indicated times. **(D)** 10-d-old (for seedlings) or five to six-week-old (for mature leaves, roots, flowers, stems) Arabidopsis plants were sampled. Primer pairs used are listed in Table S2.
