# Supplementary material for: A U-Box E3 Ubiquitin Ligase, PUB20, Interacts with the Arabidopsis G-Protein β Subunit, AGB1
Source: PLoS One. 2012 Nov 15;7(11):e49207. doi: 10.1371/journal.pone.0049207 (PMC3499536; doi:10.1371/journal.pone.0049207)
Supplement: Figure S4 — pub20 mutant. (A) A schematic representation of pub20 allele, with the T-DNA insertion shown as an inverted triangle. Primer pairs used for genomic PCR analysis (B) and RT-PCR analysis (C) are shown with arrows. The U-box domain (black box) and the ARM repeat domain (gray box) were predicted by comparing the amino acid sequence of PUB20 with PUBs described by Trujillo, Ichimura, Casais and Shirasu (Current Biology 18∶1396-1401, 2008). (B) Confirmation of T-DNA insertion in the pub20 mutant by genomic PCR. The sequences of primers specific to PUB20 ORF (FW2 and RV2) is shown in Table S3. The sequence of the T-DNA-specific primer LB3 was obtained from the website of The Nottingham Arabidopsis Stock Centre (NASC; http://arabidopsis.info/). (C) Expression analysis of UBQ5 and PUB20 by RT-PCR. Primers are listed in Table S2 and S3. (PDF) [file pone.0049207.s004.pdf]

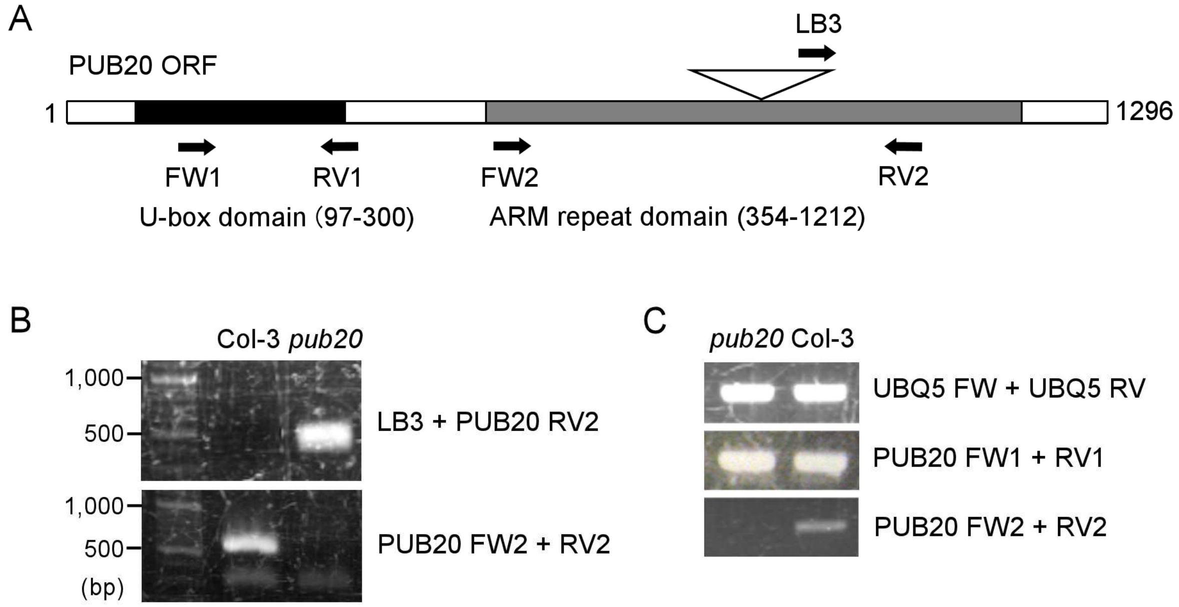

Figure S4. *pub20* mutant. (A) A schematic representation of *pub20* allele, with the T-DNA insertion shown as an inverted triangle. Primer pairs used for genomic PCR analysis (B) and RT-PCR analysis (C) are shown with arrows. The U-box domain (black box) and the ARM repeat domain (gray box) were predicted by comparing the amino acid sequence of PUB20 with PUBs described by Trujillo, Ichimura, Casais and Shirasu (Current Biology 18: 1396-1401, 2008). **(B)** Confirmation of T-DNA insertion in the *pub20* mutant by genomic PCR. The sequences of primers specific to *PUB20* ORF (FW2 and RV2) is shown in Table S3. The sequence of the T-DNA-specific primer LB3 was obtained from the website of The Nottingham Arabidopsis Stock Centre (NASC; http://arabidopsis.info/). **(C)** Expression analysis of *UBQ5* and *PUB20* by RT-PCR. Primers are listed in Table S2 and S3.
