# Supplementary material for: A U-Box E3 Ubiquitin Ligase, PUB20, Interacts with the Arabidopsis G-Protein β Subunit, AGB1
Source: PLoS One. 2012 Nov 15;7(11):e49207. doi: 10.1371/journal.pone.0049207 (PMC3499536; doi:10.1371/journal.pone.0049207)
Supplement: Table S1 — Primers used for making constructs in this work. (PDF) [file pone.0049207.s005.pdf]

Table S1. Primers used for making constructs in this work.

	Primer sequences	Names of constructs
PUB20 *Xba*IFW	CCGTCTAGAATGGGACTTTCATTGAGAGT	pBS-35S:PUB20-VC80
PUB20 *Sal*I stop *Sac*I RV	CGGAGCTCAGCGTCGACAAAATGGTTTCTTAAC ATG	pBS-35S:PUB20-VC80
PUB20 *Eco*RI *Xba*I FW	CCGAATTCTCTAGAATGGGACTTTCATTGAGAG T	pGAD-PUB20ΔARM
PUB20 ΔARM SalI XhoI RV	GGGCTCGAGCCGTCGACTCACGGGTACACGAG GCGTCGGG	pGAD-PUB20ΔARM
PUB21 promoter 5' *Hind*III FW	CCGAAGCTTCCATGTCATAATGTATTCGAG	pBI121-PUB21pro:*GUS*
PUB21 promoter 3' *Kpn*I RV	CCGGTACCGTTTTGTAAAATGCAAAAATAG	pBI121-PUB21pro:*GUS*
PUB20 *Sal*I FW	CCGGTCGACATGGGACTTTCATTGAGAGT	pET-32b(+)-PUB20
PUB20 *Xho*I RV	CCGCTCGAGTTAAAATGGTTTCTTAACAT	pET-32b(+)-PUB20
AGB1 BiFC 5' *Kpn*I FW	CCGGTACCGGAATGTCTGTCTCCGAGC	pBS-35S:AGB1-VN154
AGB1 BiFC 3' *Spe*I RV	CCACTAGTAATCACTCTCCTGGTCCTCC	pBS-35S:AGB1-VN154
AGB1 Y2H 5' *Eco*RI FW	CCGGAATTCATGTCTGTCTCCGAGCTCAA	pGBKT7-AGB1
AGB1 Y2H 3' *Bam*HI RV	CCGGGATCCTCAAATCACTCTCCTGTGTC	pGBKT7-AGB1
AGG1 BiFC 5' *Kpn*I FW	GGGGTACCATGCGAGAGGAAACTGTGG	pBS-35S:AGG1-VC80
AGG1 BiFC 3 *Spe*I RV'	CCACTAGTAAGTATTAAGCATCTGCAGCC	pBS-35S:AGG1-VC80
