# Supplementary material for: A U-Box E3 Ubiquitin Ligase, PUB20, Interacts with the Arabidopsis G-Protein β Subunit, AGB1
Source: PLoS One. 2012 Nov 15;7(11):e49207. doi: 10.1371/journal.pone.0049207 (PMC3499536; doi:10.1371/journal.pone.0049207)
Supplement: Table S2 — Primers used in real-time RT-PCR analysis. (PDF) [file pone.0049207.s006.pdf]

Table S2. Primers used in RT-PCR analysis.

	Primer sequences
PUB20 FW1	CGGGGATCACTTACGATCGCGA
PUB20 RV1	GTGACGACCCGCACCAACCT
PUB21 FW	TCCGACGCCACGTGTCCCTT
PUB21 RV	TCTCGTAATCCCCACGCCGC
