# Supplementary material for: A U-Box E3 Ubiquitin Ligase, PUB20, Interacts with the Arabidopsis G-Protein β Subunit, AGB1
Source: PLoS One. 2012 Nov 15;7(11):e49207. doi: 10.1371/journal.pone.0049207 (PMC3499536; doi:10.1371/journal.pone.0049207)
Supplement: Table S3 — Primers used in genotyping and RT-PCR analysis of the pub20 mutant (Fig. S4B, C). (PDF) [file pone.0049207.s007.pdf]

Table S3. Primers used in genotyping and RT-PCR analysis of the *pub20* mutant (Figure S4B, C).

	**Primer sequences**
PUB20 FW2	GTGACCAGTCATCAAGTCTCCG
PUB20 RV2	GCAAACGCTGTTTTCTGAATC
