# Supplementary material for: A U-Box E3 Ubiquitin Ligase, PUB20, Interacts with the Arabidopsis G-Protein β Subunit, AGB1
Source: PLoS One. 2012 Nov 15;7(11):e49207. doi: 10.1371/journal.pone.0049207 (PMC3499536; doi:10.1371/journal.pone.0049207)
Supplement: Table S4 — Effects of plant hormones on germination rate. (PDF) [file pone.0049207.s008.pdf]

Table S4. Effects of plant hormones on germination rate.

Treatment	Effect on germination rate[1]			Reference
	agb1	*pub20*	WT	
1 μM ABA	--	-	-	8
10 ppb brassinolide	0	+	+	3
100 mM NaCl		-	-	
1 μM flg22		0	0	
5 μM brassinazole		-	-	

[1]0, little effect; +, promoted; -, retarded; --, strongly retarded
